# Zeolite-Based Materials for the Catalytic Oxidation of VOCs: A Mini Review

**DOI:** 10.3389/fchem.2021.751581

**Published:** 2021-10-04

**Authors:** Tianshan Xue, Li Yang

**Affiliations:** Institute of Atmospheric Environment, Chinese Research Academy of Environmental Sciences, Beijing, China

**Keywords:** zeolite, VOCs, catalytic, oxidation, monolithic

## Abstract

Catalysts for VOCs combustion have been widely studied and zeolite-based materials and have been structured to meet the need of particle use in this field. This review summarized several new trends in zeolite-based catalysts for VOCs catalytic oxidation. Intensive effort has been devoted to the optimization of composition and structure of catalysts, abatement of CVOCs, design of zeolite-based monolithic catalysts and adsorbent/catalyst bi-functional material. The suggestions for further work here presented are put forward based on the collation of recently published papers.

## Introduction

Volatile organic compounds (VOCs) as common precursors for PM_2.5_(inhalable particles with diameters that are generally 2.5 μm and smaller) and O_3_ which have been highly concerned by researchers. They are general representatives of organic compounds with boiling points below 250°C at 101.325 kPa (He C. et al., 2019). The catalytic oxidation of VOCs is considered an efficient method due to the reduced need for fuel and toxic by-product (Liotta, 2010). In the recent decade, zeolites-based materials have been widely applied in exhaust purification and greenhouse gas adsorption (Megías-Sayago et al., 2019).

In general, zeolites can be divided into macroporous (>50 nm), mesoporous (2–50 nm), and microporous (<2 nm) zeolites, according to the pore size. These particularities directly determine the absorption and catalytic performance of the specific type of zeolite (Chandak et al., 1997). The kinetic diameter of VOCs molecules is usually less than 1 nm. Only when the pore size is larger than the molecular dynamics size of VOCs, they will then enter the pore canal of zeolite, and will be subjected to pore wall superposition or capillary cohesion. This increases the adsorption capacity, further expanding the catalytic reaction rate (Kim and Ahn, 2012). The large specific surface area and suitable pore structure of zeolite materials can reduce the agglomeration of active components and enhance the mechanical strength of materials. In addition, the large number of acidic sites on the surface of zeolites also triggered certain catalytic activity. These advantages reminded researchers to structure zeolite-based catalysts for VOCs combustion.

At present, there are mainly four mechanisms used to explain the surface adsorption, activation and reaction of reactants and catalysts, namely (i) power−low (P−L) mechanism; (ii) Eley−Rideal (E−R) mechanism; (iii) Langmuir Hinshelwood (L−h) mechanism; and (iv) Mars vankrevelen (MVK) mechanism (Behar et al., 2015). The kinetic model can be established by analyzing the conversion data obtained after varying the concentration of VOCs and oxygen in the reaction temperature. The reaction rates of VOCs under different reaction conditions were calculated according to the experimental data, and fitted with different models. The closer the coefficient of determination R^2^ (coefficient correlation) was to 1, the more suitable the model was. Besides, experimental methods such as H_2_-TPR, XPS, TGA and isotope-O_2_ can also be used to determine the reaction kinetics mechanism (Mi et al., 2021).

In most studies, the Mars-van Krevelen model was deemed as the most suitable model for VOCs oxidation. This method is based on the idea that the adsorbed VOCs react with the lattice oxygen of the catalyst, rather than the oxygen in the gas phase. The MVK model assumes that, when the reactant molecules react with the oxygen-rich part of the catalyst, the active components in the reaction can undergo alternating reduction and oxidation reactions. In this case, the oxygen in the reaction process can be chemisorbed or lattice O_2_. The model assumes that the oxidation of VOCs is divided into two steps. The first step is that the adsorbed VOCs combine with the lattice oxygen on the catalyst surface and are oxidized to CO_2_ and H_2_O. At the same time, oxygen vacancies are generated on the catalyst surface and reduced, causing metal oxides in the catalyst to be reduced as well. In the second step, the reduced metal oxide in the catalyst is oxidized again by external O_2_ to fill the oxygen vacancy in order to continue the first step reaction (Rodrigues, 2008)

In this review, we summarized the forefront researches of zeolite-based catalysts for VOCs combustion, which provides a reference to design novel VOC oxidation materials.

## Preparation and Application of Zeolites and Its Composite Materials

The catalytic activity of zeolites catalysts is highly concerned by the acidic sites on the inner pore surface. Single phase zeolites with abundant acidic sites usually perform better in the catalytic process. When the acidity is strong, carbon deposition is likely to occur on the catalyst surface, which affects the catalytic activity (He et al., 2009). ZSM-5 (Zeolite Socony Mobil–5) and SBA-15 (Santa Barbara Amorphous of number 15) have moderate acidity and higher stability, but their catalytic activity is relatively low. However, zeolite catalysts have no advantages over noble metals and transition metal oxides when applied for VOCs oxidation reaction. Therefore, the design of two-phase zeolite materials as catalyst may not have any evident effect on the improvement of catalytic activity. Researchers prefer to use the unique pore structure of molecular sieves to support precious metals for VOCs combustion. In all the previous studies, the main indicator of catalytic activity is temperature at which 50, 90 or 100% conversion of VOCs have achieved, noted as T_50_, T_90_ and T_100_, respectively.

When a noble metal is loaded on the zeolite, the catalytic activity is highly associated with the type, structure and dispersion of this noble metal. Pd/BEA and Pd/FAU zeolites were found to be powerful catalysts for the total oxidation of toluene and propene (Tidahy et al., 2007). Cations could improve the dispersion of noble metal species but decrease the surface area and the micropore volume. Zhang et al. (2018) examined a Pt loaded hierarchical porous MOR (Mordenite) zeolite obtained by a facile acid and alkali post-treatment strategy. The substance exhibited superior performance for toluene catalytic oxidation. In this case, T_90_ of as-synthesize catalyst over toluene is only 190°C (1,000 ppm toluene in air; WHSV, Weight Hourly Space Velocity = 60,000 ml/(gH)). The neural network model coupled with genetic algorithm was employed to find the optimal transition metal to assist the Ag-zeolite catalyst for VOCs abatement (Izadkhah et al., 2012). It was found that the addition of Fe, Ni and V was more active for ethyl acetate oxidation than in untreated Ag-ZSM-5.

Almost all the transition metal oxides have the potential for VOCs catalytic oxidation. Cu, Mn, Co and Fe are the common transition metals loaded on zeolite to trigger this process. Soylu et al. (2010) examined these four kinds of oxides supported on natural clinoptilolite-type zeolites to completely degrade toluene. They ordered the activity of fabricated catalysts as: MnO_2_ >CuO > Co_3_O_4_ >Fe_2_O_3_ and attributed this phenomenon to the surface Lewis acidity, which played a dominant role in determining the catalytic activity at low temperature.

**FIGURE 1:**
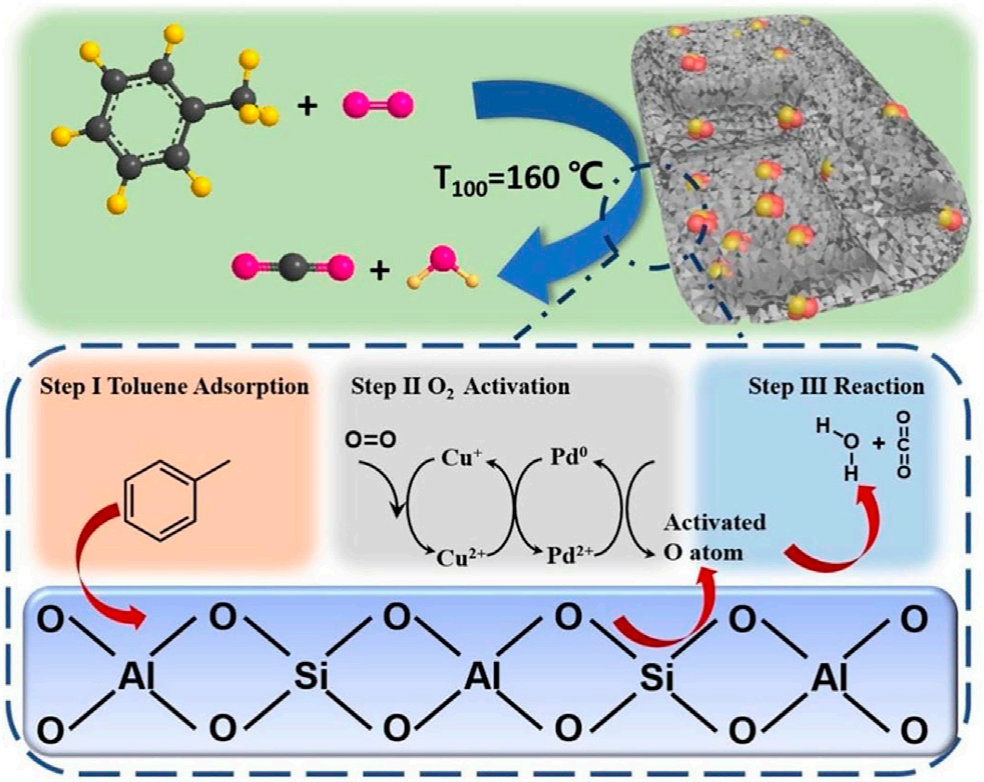
Mechanism of toluene degradation on the surface of PdCu alloy supported on mesoporous ZSM-5 (He et al., 2019).

Researchers have conducted multiple studies on the structural control of oxides or mixed oxides to achieve higher activity. In order to obtain highly uniformed nana-structure, surfactant (e.g., rich-hydroxyl) complexing agents were introduced in the preparation process of materials. Zhang C. et al. (2020) compared the catalytic activity of Co_3_O_4_ nanoparticles onto HZSM-5 with the addition of β-cyclodextrin, sorbitol and citric acid, respectively. The results showed that the Co_3_O_4_/HZSM-5/β-cyclodextrin catalyst with increased low-temperature reducibility and more plentiful surface acid sites were more suitable for toluene oxidation. The T_90_ value for toluene was measured at 288°C (1,000 ppm toluene, 100 ml/ min). Liu F. et al. (2019) recently intercalated nanosized Pt into single-layer sheets of ZSM-5 to form a sandwich-structured catalyst for toluene oxidation. The unique construction ensured a better dispersion and higher thermal stability of Pt. It made Pt/ZSM-5 displayed higher catalytic activity—around 98% of toluene could be totally transferred to CO_2_ and H_2_O at 176°C (1,000 ppm, SV = 60,000 ml/(g h)). The use of carbon material as sacrificial agent is a novel route to build highly dispersed surface structure of catalysts. The loss of carbon black encapsulated by zeolite as a route to form PdCu alloy supported on mesoporous ZSM-5 zeolite (He J. et al., 2019). The alloying process of Cu and Pd resulted in a better activity for toluene oxidation (T_90_ = 152°C, 50 ppm toluene, WHSV = 36000 ml/(g h)) is shown in Figure 1.

The combination of perovskite and zeolite was studied by Zang et al. (2019). The structure and morphology of three-dimensionally ordered macroporous and nanoparticle La_0.8_Ce_0.2_MnO_3_ significantly enhanced the toluene oxidation active associated with the specific surface area of the catalyst. The T_50_ and T_90_ value were estimated as 147°C and 217°C. Single atom catalyst is another Frontier of catalyst preparation and application for VOCs combustion (Wang Y. et al., 2020; Zhang et al., 2021). Nozaki et al. (2002) put forward a novel method to prepare a series of single-site catalysts that have isolated iron centers onto SBA-15 by grafting reactions of the Fe[OSi(OtBu)_3_]_3_ (THF) with SBA-15 in dry hexane.

**FIGURE 2:**
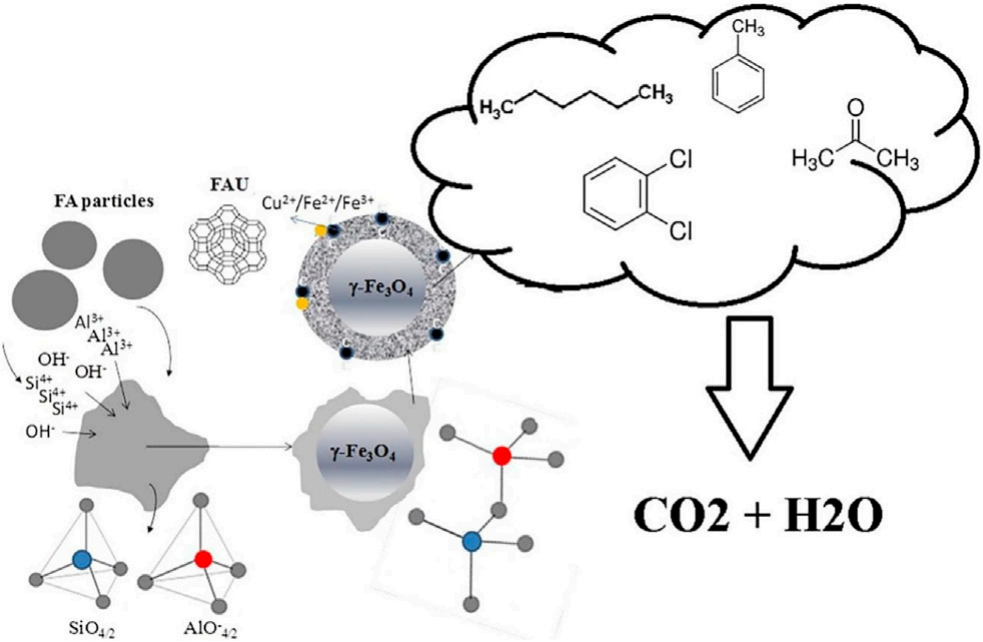
Schematic diagram of zeolite prepared from fly ash and its catalytic performance for VOCs (Boycheva et al., 2019).

The high cost of raw material and the complexity of the method are the main constraints for the large-scale use of zeolite. This is why the idea of cheap or waste materials-derived zeolite was brought up. It is worth noting that these kinds of zeolites need to overcome the influence of impurities in order to become active and stable catalysts. Boycheva et al. (2019) used fly ash gathered from thermal power plants to synthesize Na-Y zeolite as shown in Figure 2. The catalytic oxidation of n-hexane, acetone, toluene, 1,2 dichlorobenzene was operated on the Cu-modified fly ash-derived zeolite. Cr-loaded NaY zeolite obtained by bio recovery of chromium from water for ethyl acetate, ethanol and toluene oxidation was reported by Silva et al. (2011). For all VOC tested, the Cr-loaded zeolite performed well and presented satisfactory CO_2_ selectivity. Authors believed that the presence of Cr shifted the reaction pathways, leading to enhancement of CO_2_ selectivity.

## Zeolite-Based Catalysts for CVOCs Removal

Along with the refinement studies, the control of chlorinated volatile organic compounds (CVOCs) has received a lot of attention from scholars due to their high stability, poor reactivity and high toxicity (Li et al., 2020). CB (C_6_H_5_Cl), DCE (CH_2_ClCH_2_Cl), DCM (CH_2_Cl_2_), and TCE (C_2_HCl_3_) are the typical CVOCs discharged by industrial sources. The main difficulties in CVOCs catalytic combustion process are the formation of harmful and incomplete combustion products (such as COCl_2_) and the deactivation of catalysts due to coking, carbon deposition and chlorination.

Zeolite materials have a large number of acidic sites. The synergetic effect of Lewis and Bronsted acid sites can effectively improve the chlorobenzene degradation activity and stability. Among them, the introduction of Lewis acid sites promotes the activation of lattice oxygen, improves the redox performance, and is conducive to the oxidation of organic matter and the oxidative removal of adsorbed chlorine. The existence of Bronsted acid site changes the degradation path of chlorobenzene from oxidation to hydrolysis path, promotes the emission of Cl ions in the form of HCl, fundamentally inhibits the formation of Cl_2_, and reduces the selectivity of chlorine containing by-products and Cl_2_ (Lv et al., 2021).

Variations of zeolite were designed as active ingredient or carrier of catalysts for the purpose of CVOCs abatement and achieved remarkable results. The catalytic activity of HY zeolite can be improved by adjusting the ratio of silicon to aluminum (Lopez-Fonseca et al., 2003). De-alumination by ammonium hexafluoro silicate treatment increased the number of strong Brønsted sites. DCE could be totally converted at 350°C on modified HY zeolite, and the T_50_ values of DCM and TCE were 345°C and 465°C, respectively (1,000 ppm CVOCs, GHSV = 15,000 h^−1^).

To reach better catalytic activity, noble metals such as Au, Ag, Pt, and Pb with great properties were also loaded on zeolite for CVOCs removal. Wang Z. et. al. (2020) investigated a bimetallic Pt-Ru supported on HZSM-5 for CB (C_6_H_5_Cl) catalytic removal. Pt_0.5_Ru_0.5_/m-HZ catalyst could convert 50% CB at 234°C (1,000 ppm CB/dry air, GHSV = 40,000 ml/ gh), and the high selectivity for less toxic products (CO_2_ and HCl) and scarce formation of chlorinated byproduct during 50 h test.

The combination of transition metals combined with zeolites have been widely used to catalytic degrade CVOCs as well. Gonzalez-Prior and co-workers conducted a study of a Co_3_O_4_/SBA-15 structured catalyst for DCE oxidation (Gonzalez-Prior et al., 2016). They have verified that SBA-15 provided surface OH groups of SBA-15 as grafting points in the impregnation process and regulated the dimensions of Co_3_O_4_ nanoparticles by its mesopore structure. Cobalt-loading affected the acidity species and strength of catalysts by adding Lewis acidic sites and reducing the medium-weak Brønsted acidic sites. The T_50_ value of 40Co/SBA was 330^o^C and lattice oxygen species in the catalyst were associated with Cl-VOC combustion following a Mars-van Krevelen mechanism. (Ce,Cr)_x_O_2_/HZSM–5 composite materials were prepared by Yang et al. (2017) and performed remarkably in DCE degradation. The HZSM-5 provided large amount of acid sites to promote the adsorption and dehydrochlorination of DCE (CH_2_ClCH_2_Cl). After that, the (Ce,Cr)_x_O_2_ could oxidize the by-product to CO_2_ quickly and thoroughly. (Ce,Cr)_x_O_2_/HZSM-5 after long-term test could be recovered by 400°C calcination. Blanch-Raga et al. (2016) fabricated a Cu-exchanged zeolite for TCE oxidation and the T_50_/T_90_ values registered by them were 310°C and 360°C, respectively. They proposed the oxidative properties of Cu could avoid the formation of coke and protect the acid sites on the surface of the zeolite. The activity and selectivity of as-synthesized catalyst in TCE oxidation process was associated with the acidity, redox properties and metal-zeolite interaction. Gołąbek et al. (2019) studied the activity of Ce-BEA for the catalytic oxidation of TCE and the formation mechanism of by-products. The highly developed mesopore surface area, well-dispersed cerium and the amount of high strength Brønsted acid centres and Brønsted sites in the Ce-loaded zeolite catalyst was deemed as the crucial role of activity and selectivity.

## Zeolite-Based Monolithic Catalyst Employed for Combustion

Considering the high pressure drop and difficult separation of powder catalysts, some researchers have started to shift their interest to monolithic catalysts with zeolite components. Zeolites could be consolidated to form pellets or directly wash-coated to other carriers. In the field of VOCs combustion abatement, cordierite is the most common carrier of monolithic catalysts. It has been widely studied and practically applied because of its high strength, low volume density and low coefficient of thermal expansion (Zamaro et al., 2005; Brahmi et al., 2013).

A Pt-MFI zeolite-cordierite foam-structured catalyst was designed and adopted for toluene removal by Ribeiro and co-workers (Ribeiro et al., 2011). The open structure of cordierite foam, uniformly deposited thin layers’ catalyst, location and size of Pt joint acted to perform a good activity (T_50_≈210°C, 800 ppm toluene in 15 Lh^-1^ air flow). Silva et al. (2008) tested deep oxidation properties of isopropanol on Cu and Pt modified MFI zeolite, and achieved a rate of 100% in the conversions of CO_2_ at 160°C to Pt-MFI catalyst.

Recently, metal carriers such as iron mesh (Xue et al., 2020), stainless steel grid (Louis et al., 2001; Louis et al., 2002), and stainless-steel fiber have been widely reported as the carrier for heat treatment of exhaust gas. Monolithic catalysts with metal basal are identified as an area of future work as a result of its stable, easy shaped and highly efficient heat transfer capabilities. Hydrothermal and chemical vapor deposition (CVD) methods are most favorable for zeolite *in-situ* decoration on steel fiber.

Zhou et al. (2017) obtained Cu modified catalysts supported on Linde Type A zeolite membrane/paper-like stainless steel fibers (LTA/PSSF) using CVD method. The selected 10% Cu/LTA/PSSF catalyst performed well towards acetone oxidation with T_90_ values at 300°C (1,500 ppm in air). They verified that the CVD method has the potential to obtain highly uniformed CuO and zeolite nonparticipants onto PSSF basal. Liu G. et al. (2019) synthesized Cu and Mn mixed oxides modified beta zeolite coated on PSSF for toluene combustion. The typical truncated bipyramidal shape of beta zeolite on PSSF was obtained via wet lay-up papermaking and sintering process. Then, Cu and Mn species were introduced by an incipient wetness impregnating method. Results showed that the catalytic activities over toluene increased as the Mn content increased as well. The lowest T_90_ value was 302°C (1,000 ppm toluene in air, 200 ml/ min flow rate) presented by the CuMn_3_/Beta/PSSF catalyst.

PSSF based monolithic catalysts have also been investigated in CVOCs removal. Zhang Y. et al. (2020) developed Cr modified ZSM-5 as the active phase onto PSSF membranes and studied the mechanism of TCE combustion (T_90_ = 300°C, 1,500 ppm in air). The Mars-van Krevelen kinetic model could fit well with the TCE degradation data and the reaction activation energy for the surface oxidation/ reduction reaction were 64.57 kJ/ mol and 122.25 kJ/ mol, respectively.

## Zeolite-Based Dual Functional Adsorbent/Catalyst System for VOCs

VOCs are also emitted from vehicles and in-door cooking. In these cases, the size of treatment facilities is limited to small cage. Therefore, the combination of adsorbents and catalysts is meaningful. Yu et al. (2020) reported a novel ferrisilicate MEL zeolite for cleaning cooking oil fumes with non-methane hydrocarbon. The hydrophobic Si-Fe framework of the catalyst enhanced their hydrophobic feature due to the substitution of Al by Fe. Various VOCs with different molecular dimensions could be firstly adsorbed onto the catalysts’ surface and immediately oxidized by the nanorod-stacking FeO_x_. Jang et al. (2021) studied Cu-impregnated BEA zeolite for aromatic adsorption and oxidation applied to control of vehicles’ waste gas. Dispersed Cu ions allowed the preferential adsorption of HCs, and the nanosized CuO particles on the exterior surface could oxidize HCs starting from 210°C. The key to achieve high removal efficiency was preferential adsorption of propene under wet conditions.

In large-scale industrial processes, adsorption and catalytic oxidation are the two main stages of VOCs removal. With the aim of shortening the working process and minimizing energy consumption, an integrated material combining adsorption and combustion for industrial use was proposed. Baek et al. (2004) tested the adsorption/desorption and catalytic performance of hydrophobic Y zeolite materials introduced with different amount of Ag, Mn, Fe, Co, Cr, Cu, Ni, Pt, and Pb. The Ag/HY was selected as the best candidate for the dual functional system. The large adsorption capacity and low desorption temperature in the narrow desorption window guaranteed the continuous adsorption-regeneration cycling. Also, the toluene activity (T_100_ = 290°C, 1,000 ppm, SV = 6,000 h^−1^) and MEK (T_100_ = 290°C, 1,000 ppm, SV = 6,000 h^−1^) oxidation was enhanced by increasing the Ag loading.

Overall, adsorption and catalytic abatement is a new trend in civil or industrial treat of VOCs in which zeolite-based material could give full play to its specialty. Researchers should pay more attention on the economic efficiency and feasibility of this method, and keep studying by-products and the resistance of water vapor and high temperature.

## Discussion

Zeolites have been regarded as a future key components in VOCs combustion catalyst, and many studies have been conducted around this topic. The focus of this work is the optimization of catalyst performance, the reduction of preparation costs and the popularization of its practical application. In recent years, optimization of composition and structure of catalysts have become more diversified and delicately for acquire higher activity. Target pollutants gradually expanded from commonly benzene series VOCs to CVOCs. To meet the need of more practical applications, powder catalysts are assembled or coated to different basal to form monolithic catalysts. Adsorbent/catalyst bi-functional material was reported and attempted to solve the VOCs oxidation in cook, vehicle or industrial situations.

Besides, some new frontiers of zeolite-based catalysts have been recently discovered but few studies about it have been published. For instance, to meet the worldwide demand to reduce greenhouse gas emission and achieving carbon neutrality, the oxidation of VOCs and the adsorption of CO_2_ emitted after VOCs catalytic combustion could be successively achieved by zeolite (Mp et al., 2020). The mixture effect with different VOCs under oxidation was studied by Beauchet et al. (2010) and an inhibiting effect of the o-xylene on the isopropanol oxidation was observed. This is because the adsorbed aromatic VOCs near the apertures of the NaX supercages limit the access of isopropanol to the basic active sites.

We need to widen the studies around the mechanism of VOCs oxidation induced by zeolites, and explore the ways to deal with the water and high temperature conditions that may be encountered in practical applications. Besides, the simultaneous removal of different VOCs, VOCs/NO_x_, VOCs/CO_2_ could be a more realistic problem to solve.
